# Superconducting nanowire single-photon detectors with non-periodic dielectric multilayers

**DOI:** 10.1038/srep35240

**Published:** 2016-10-24

**Authors:** Taro Yamashita, Kentaro Waki, Shigehito Miki, Robert A. Kirkwood, Robert H. Hadfield, Hirotaka Terai

**Affiliations:** 1 Advanced ICT Research Institute, National Institute of Information and Communications Technology, 588-2 Iwaoka, Nishi-ku, Kobe 651-2492, Japan; 2 Department of Materials Engineering Science, Graduate School of Engineering Science, Osaka University, 1-3 Machikaneyama-cho, Toyonaka, 560-8531, Japan; 3 School of Engineering, University of Glasgow, Glasgow, G12 8QQ, United Kingdom

## Abstract

We present superconducting nanowire single-photon detectors (SSPDs) on non-periodic dielectric multilayers, which enable us to design a variety of wavelength dependences of optical absorptance by optimizing the dielectric multilayer. By adopting a robust simulation to optimize the dielectric multilayer, we designed three types of SSPDs with target wavelengths of 500 nm, 800 nm, and telecom range respectively. We fabricated SSPDs based on the optimized designs for 500 and 800 nm, and evaluated the system detection efficiency at various wavelengths. The results obtained confirm that the designed SSPDs with non-periodic dielectric multilayers worked well. This versatile device structure can be effective for multidisciplinary applications in fields such as the life sciences and remote sensing that require high efficiency over a precise spectral range and strong signal rejection at other wavelengths.

In recent years, superconducting nanowire single-photon detectors (SSPDs or SNSPDs) have emerged as promising single-photon detectors due to their many attractive features, such as high detection efficiency, low dark count, and small timing jitter^1^,^2^,^3^. Furthermore, SSPDs have an intrinsic broadband sensitivity from the visible to the mid-infrared wavelength region and have already been applied with considerable impact in research fields such as quantum information^4^, quantum optics^5^, and free space laser communication^6^. For these applications, SSPDs need to have high efficiency at their respective target wavelengths, and recent progress has enabled system detection efficiency (SDE) greater than ~70% at wavelengths from 500 to 1600 nm by applying simple resonant cavity structures to enhance the optical absorptance in the meandering superconducting nanowire^7^,^8^,^9^,^10^,^11^,^12^. These simple cavity structures consist of the following layers in the following order: single or double dielectric resonant layers with thicknesses set to approximately quarter wavelength, a superconducting meandering nanowire, and a mirror layer made of a metallic material or dielectric multilayers. This structure is relatively simple and can effectively achieve high absorptance at the target wavelength, and the wavelength dependencies of absorptance show a single peak structure. However, detectors with high efficiency over a carefully controlled spectral range, with rejection at other wavelengths, are highly desirable for emerging applications in the life sciences and atmospheric remote sensing. For example, in the visible to near-infrared range, applications such as fluorescence correlation spectroscopy (FCS)^13^,^14^, Raman spectroscopy^15^,^16^, and multispectral single-photon light detection and ranging (LIDAR) for forestry and agriculture^17^, appropriately tailored SSPDs will have superior characteristics to off-the-shelf silicon avalanche photodiodes^18^.

In this paper, we propose and demonstrate a device structure of SSPD with a *non-periodic* dielectric multilayer (DML), which enables a variety of designs for the wavelength dependences of the optical absorptance in a superconducting nanowire. Figure 1 shows a schematic of the SSPD structure on a non-periodic DML. In this structure, two different dielectric materials with different refractive indices are placed on the substrate, and the superconducting nanowire is located on the DML. By optimizing the thicknesses of each dielectric layer in the DML, one can design the required wavelength dependence of the optical absorptance in the superconducting nanowire. Advantage of the SSPD with non-periodic DML is that one can achieve the various wavelength dependences such as wider or narrower bandwidth and/or an intrinsic bandpass filter to minimize the effect of blackbody radiation, pump- or stray light without changing the basic device structure. Although periodic DMLs have been used for SSPDs as a mirror for shorter wavelengths simply to enhance the optical absorptance so far^10^,^11^, the non-periodic DML structures shown in the present paper could provide new functionalities to the wavelength dependence of the optical absorptance of SSPDs. In this study, SiO_2_ and TiO_2_ were used as dielectric materials, and NbN was used for the superconducting nanowire. The incident photons enter the device from the front side. For this structure, we made examples of the desired wavelength dependences of the optical absorptance by optimizing the design of the DML as described below.

## Results

### Numerical simulation

**Figure 1:**
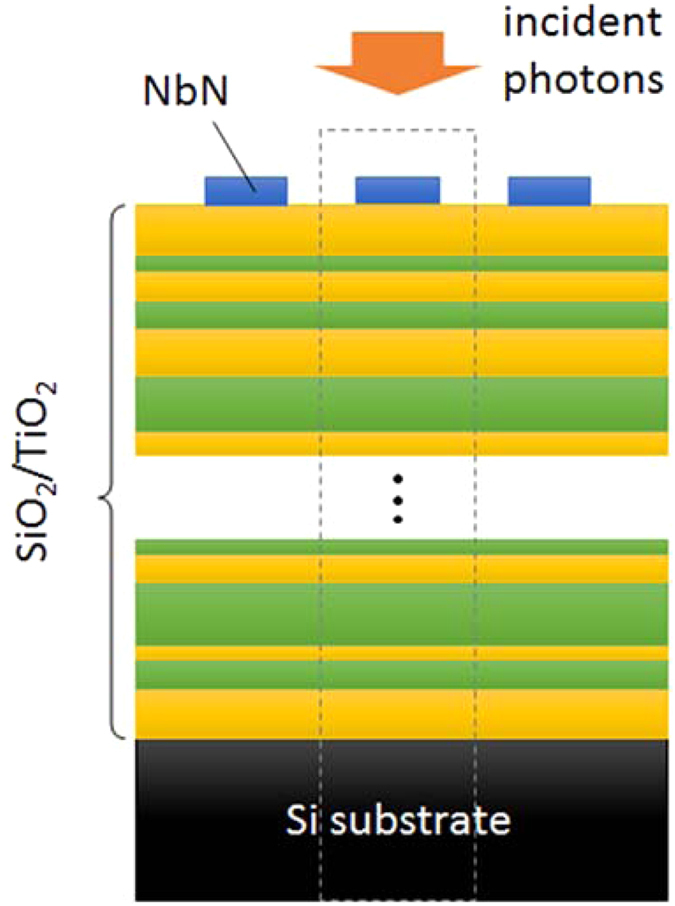
Schematic of an SSPD with a non-periodic dielectric multilayer. The area surrounded by the dashed line indicates a unit cell in the finite-element analysis.

A straightforward way to optimize the DML design in SSPD is to perform a numerical simulation of the optical absorptance in the nanowire with changing thicknesses of each layer in the DML using finite element analysis (FEA) and so on^19^,^20^,^21^. However, it requires substantial computational time and power to solve for the absorptance in the nanowire for multiple combinations of DML with various thicknesses by using the FEA. Therefore, we performed the following efficient processes to optimize the DML design. First, the optimization of the DML under the unpatterned NbN thin film was performed using a commercial optical thin film software package (Essential Macleod, Thin Film Center Inc.) based on the transfer matrix method^22^. The software can automatically optimize the thicknesses of each SiO_2_ and TiO_2_ layer to realize the desired wavelength dependence of the absorptance in the NbN film by setting the specification requirements. Meanwhile, it is possible to show the different wavelength dependences of a meandering NbN nanowire from that of the unpatterned film even on the same DML design. Therefore, we confirmed the optical absorptance of the NbN nanowire on the DML using the FEA software, COMSOL 5.2 with RF module (COMSOL Inc.)^19^,^20^. Here we assumed a two-dimensional stack structure with the grid NbN layer, as shown in Fig. 1, and calculated the wavelength dependence of the optical absorptance in the NbN nanowire on the DML obtained from the previous optimization process. Using the FEA, we calculated the dependence of the optical absorptance on the polarization of the incident light, e.g. the TE (parallel to the nanowire) and TM (perpendicular to the nanowire) modes^19^,^20^.

**Figure 2:**
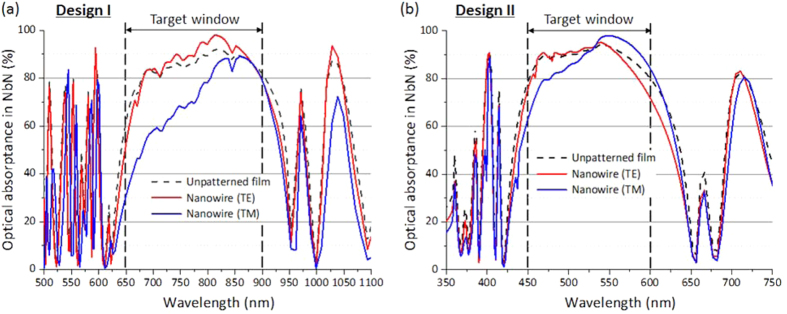
Calculated wavelength dependences of the optical absorptance in NbN for a non-periodic DML. Black dashed and red (blue) solid curves indicate the absorptance in the NbN unpatterned film and NbN nanowire for the TE (TM) mode, respectively. (**a**) Design I: the target window is 650–900 nm. (**b**) Design II: the target window is 450–600 nm.

According to the optimization process described above, we designed two types of SSPDs on the non-periodic DML with a high optical absorptance range near 800 nm (Design I) and 500 nm (Design II), which are important wavelengths for applications in quantum optics^5^ and life sciences^13^,^14^, respectively. For both designs, we fixed the thickness of the NbN layer to 10 nm. Figure 2(a) shows the wavelength dependence of the optical absorptance for Design I. In the DML optimization process for Design I, we imposed a target condition where the optical absorptance attempts to approach 100% in the wavelength range of 650–900 nm under the condition that the total layer number of the DML is less than 40. As a result, we obtained an optimized DML design with 27 layers and a total thickness of 5.8 μm. The thickness of each layer in the DML was 2–561 nm. Then, we calculated and checked the optical absorptance in the NbN nanowire using the FEA. Here we assumed that the line width and pitch of the nanowire in the unit cell were 120 and 200 nm, respectively. In Fig. 2(a), the black dashed curve indicates the obtained absorptance spectrum of the unpatterned NbN film on the optimized DML. High optical absorptance over 60% was realized for the target wavelengths of 650–900 nm for the optimized DML. The red (blue) curve in Fig. 2(a) indicates the optical absorptance in the NbN nanowire for incident light with the TE (TM) mode calculated by FEA. As shown in the figure, the optical absorptance for the TE mode is always higher than that for the TM mode in the target window, and the absorptance spectrum for the TE mode shows an excellent absorptance of over 90% near the wavelength of 800 nm.

Figure 2(b) shows the optimized wavelength dependence of the absorptance for Design II, which was obtained for the target wavelength range of 450–600 nm. As shown in the figure (black dashed curve), a high absorptance of over 80% was realized for the unpatterned NbN film at the target wavelength. From the optimization process, the thickness of each layer in the DML was 10–175 nm, and the total number of layers and thickness of the DML were 29 layers and 2.0 μm, respectively. Using the obtained thicknesses of the SiO_2_ and TiO_2_ layers, we calculated the absorptance in the NbN nanowire assuming that the line and pitch of the unit cell were 150 and 250 nm, respectively. As shown in Fig. 2(b), the absorptance for the TE mode (red curve) is higher than that of the TM mode (blue curve) below a wavelength of 530 nm and vice versa over 530 nm in the target window, and a high absorptance of ~80% was realized near a wavelength of 500 nm for the TE mode.

### Fabrication and experimental setup

Based on Designs I and II, we fabricated the SSPDs with non-periodic DML. The SiO_2_ and TiO_2_ films with the designed thicknesses were sputtered on the Si substrate for the DML prior to the fabrication of both devices, and then a 10-nm-thick NbN film was deposited by DC reactive sputtering at the ambient substrate temperature. An arithmetic mean roughness of the DML surface was observed to be 1–2 nm by an atomic force microscope (AFM). For Design I, the NbN film was patterned to a meander structure of a nanowire with an active area of 15 × 15 μm^2^, and the nanowire width and pitch were 120 nm and 200 nm, respectively. The superconducting critical temperature was 7.5 K and the switching current was 20.8 μA at 2.2 K. For Design II, we fabricated an interleaved four element SSPD array to realize a high counting rate^8^. The line and pitch were 150 nm and 250 nm, respectively, which are suitable for the detection of single photons at wavelengths of 450–600 nm^10^. The active area was circular with a diameter of 35 μm for efficient optical coupling with the multimode fiber, which is necessary in life science applications, such as FCS experiments^10^,^13^,^14^. The switching currents of each element were 20.2–23.2 μA at 2.4 K and the critical temperatures were 7.5 K.

The fabricated chips were mounted in fiber-coupled packages. Our fiber-coupled package consists of fiber-holding block and chip-mounting block. We aligned these two blocks so that the incident light spot illuminates the center of the active area by monitoring the device active area and light spot from the rear side of the chip^23^. In the package for the chip based on Design I, a single-mode fiber (SMF) for the 780-nm wavelength (780HP, Thorlabs Inc.) with a core diameter of 4.4 μm was installed and fixed in front of the chip so that the distance between the tip of the SMF and the device active area was ~20 μm. The estimated diameter of the light spot at the active area is 6.3 μm. In the package for the chip based on Design II, a 50-μm-core MMF fiber (G-50/125-1005-3005-UV, Fujikura Ltd.) with spliced graded index (GRIN) lenses was installed and fixed as in Design I^10^. The GRIN lenses can focus the incident light spot to ~28 μm on the device active area. Accordingly, both packages enabled high efficiency optical coupling.

The packaged devices were installed in a 0.1-Watt Gifford-McMahon cryocooler system with the operational temperature of 2.2–2.4 K. In the cryocooler, semi-rigid cables, SMFs, and MMFs were connected to the mounted package. The semi-rigid coaxial cables were routed to a bias tee and two low-noise amplifiers outside the cryocooler at room temperature. For the measurements of SDE, continuous-wave lasers with wavelengths of 406, 520, 635, 670, 785, 808, 850, 904, and 980 nm were used as photon sources with their light power heavily attenuated so that the photon flux at the input connector of the cryostat becomes 10^4^–10^6^ photons per second. For the measurement of the device with Design I mounted in the SMF-coupled package, we used a polarization controller to control the polarization of the incident photons to maximize SDE. Although it is difficult to measure the actual polarization directly at the end of the fiber, we expect the polarization of the incident light through the SMF to be the TE mode according to the absorptance calculation shown in Fig. 2(a). The output counts from SSPD were measured using a pulse counter. SDE was determined by SDE = (*R*_output_ − *R*_DCR_)/*R*_input_, where *R*_output_ is the SSPD output pulse rate, *R*_DCR_ is the dark count rate (DCR), and *R*_input_ is the input photon rate of the cryocooler system.

## Experimental Results and Discussion

**Figure 3:**
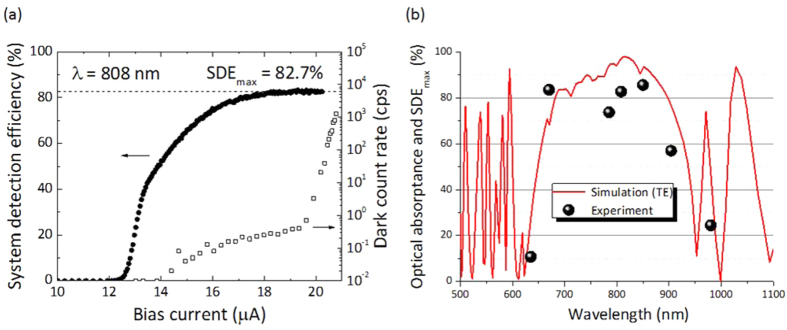
(**a**) Bias current dependences of the system detection efficiencies at a wavelength of 808 nm and the dark count rate for SSPD on DML with Design I. (**b**) Wavelength dependences of the calculated absorptance (red curve) and the measured maximum system detection efficiency (SDE_max_, circular symbols) in the bias current dependences.

Figure 3(a) shows the bias current dependences of SDE for the 808-nm-wavelength photons and the DCR at 2.2 K for the SSPD on DML with Design I. As shown in the figure, SDE reached a maximum value (SDE_max_) of 82.7% with a low DCR of less than 1 cps and showed plateau characteristics in the high bias region close to the switching current. We have examined the bias current dependencies of the SDE for various wavelength regions: 635, 670, 785, 808, 850, 904, and 980 nm, and plateau characteristics were observed for all wavelengths except 980 nm. Figure 3(b) shows the wavelength dependences of the measured SDE_max_ (circular symbols). For comparison, the wavelength dependences of the calculated optical absorptance for the TE mode of Design I (red curve) are also shown in the figure. In SSPD, SDE is a product of the coupling efficiency, the optical absorptance in the nanowire, the intrinsic pulse generation probability, and the optical loss in the system^20^. Therefore, assuming a constant optical coupling, system optical loss, and pulse generation probability over the measured wavelengths, the SDE is proportional to the optical absorptance. Indeed, the obtained SDE_max_ for each wavelength corresponded well to the calculated wavelength dependence of the optical absorptance, as shown in Fig. 3(b). The SDE_max_ showed high values of approximately 80% for wavelengths of 670–850 nm and rapidly fell below 30% outside the target window, indicating that the optimized non-periodic DML design worked as expected. The deviation of 10–20% between theory and experiment will be attributed to the optical losses in the cryocooler system, a deviation between the designed and actual size of the device and so on.

**Figure 4:**
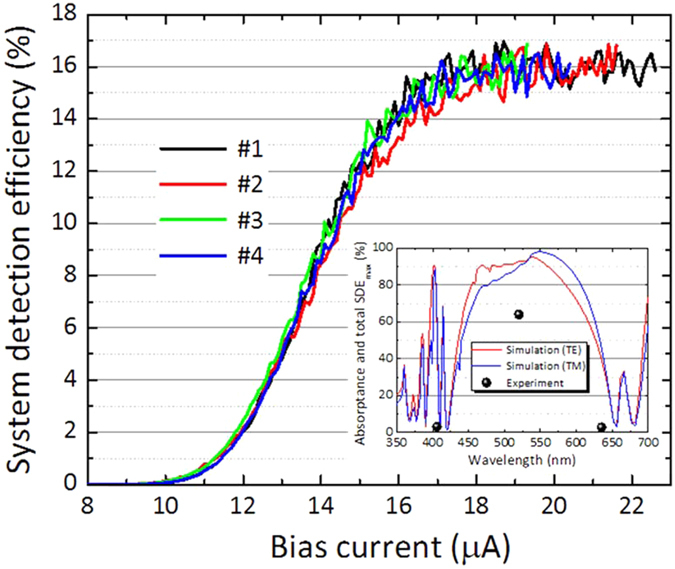
Bias current dependences of the system detection efficiency for the interleaved four-element SSPD array on the DML with Design II. The wavelength of the incident light is 520 nm. Inset: The calculated optical absorptance in the NbN nanowire (blue and red curves) and the total SDE_max_ for the wavelengths of 406, 520, and 635 nm (symbols).

Figure 4 indicates the SDEs of the four elements in the SSPD array (#1–#4) fabricated on the basis of Design II for a wavelength of 520 nm, which is within the high optical absorptance window. The inset of Fig. 4 shows a comparison of the wavelength dependencies of the calculated absorptance for Design II (red and blue curves for the TE and TM modes, respectively) and the measured SDE_max_ (symbols). As shown in Fig. 4(a), the SDEs of all the elements followed a similar curve and showed saturation close to their switching currents. The SDE_max_ in the saturated region reached approximately 16% for all the elements, resulting in a high total SDE of 64%. In the measurement, we did not observe any apparent crosstalk between the detector elements. Conversely, we observed that the total SDE_max_ for the wavelengths of 406 and 635 nm, which were outside the target window, showed very low values of ~3% as predicted by the simulation. Therefore, the SSPD array on a non-periodic DML based on Design II also worked as expected, although the obtained value of SDE_max_ at 520 nm was smaller than the calculated absorptance of approximately 90% assuming linear optical polarization. In this visible wavelength range, silicon avalanche photodiode (Si APD) has been used conventionally due to its high detection efficiency of 60–70%. Although the SDE of the presented SSPD with DML is still comparable to that of a Si SPAD, SSPDs have the additional advantage of being free from afterpulsing and have already been used successfully in FCS experiments^13^,^14^. By improving the SDE further with the DML structure presented in this work, SSPDs will have clear advantages over Si APDs and will represent an advancement in enabling technology for fluorescence experiments generally^24^,^25^. As a future work, it is also interesting to investigate how the thermal relaxation to the thick dielectric multilayer affects the detector performance metrics such as the maximum counting rate and SDE in comparison to conventional substrates.

**Figure 5:**
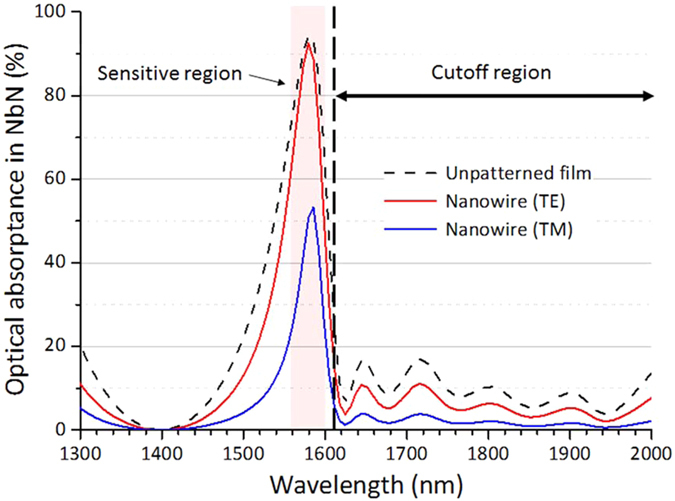
Calculated wavelength dependences of the optical absorptance in NbN for the telecom range with a cutoff. Black dashed and red (blue) solid curves indicate the absorptance in the NbN unpatterned film and NbN nanowire for the TE (TM) mode, respectively.

Moreover we are confident that our versatile non-periodic DML could be applied in the telecom range. Applications which would benefit from these next generation SSPDs would include singlet oxygen luminescence detection for laser cancer treatment^26^, fiber Raman temperature sensing^27^, differential absorption LIDAR for carbon dioxide atmospheric sensing^28^ and quantum key distribution over live real-world fiber optic networks^29^. Finally we present an example of the non-periodic DML SSPD for the telecom range. Figure 5 shows the simulation results of the optical absorptance optimized for the telecom range with cutoff over the wavelength of 1600 nm by performing the same simulation procedure of Designs I and II. In this design, the thickness of NbN was 8 nm, and the line and pitch of the nanowire are 100 nm and 200 nm, respectively. The thickness of the each layer in DML was obtained to be 5–897 nm, and the total layer number and thickness of DML were 31 layers and 6.9 μm, respectively. As shown in Fig. 5, non-periodic DML realizes a strong reduction of the optical absorptance (less than 10%) for the longer wavelengths over 1600 nm, at which the blackbody radiation enters the SSPD from room temperature through the optical fiber, whereas the high absorptance is ensured for the wavelength of 1560–1600 nm. It is expected that the extrinsic dark count caused by the blackbody radiation is strongly suppressed in the non-periodic DML SSPD^30^,^31^. Although the experimental test for this design is future work, this simulation result clearly shows the advantage of non-periodic DML SSPDs.

In conclusion, we presented SSPDs with non-periodic dielectric multilayer structures to achieve a flexible design for the visible to near infrared spectrum. We performed a robust simulation to optimize the desired optical absorptance spectrum in the superconducting nanowire. Two different spectra were designed with target windows of 650–900 and 450–600 nm. We fabricated SSPDs based on the two designs and evaluated the system detection efficiency for incident photons across a range of wavelengths. As a result, we found that the obtained detection efficiencies for each wavelength were consistent with the simulated wavelength dependencies of the optical absorptance. Furthermore, we theoretically demonstrated non-periodic DML SSPD with the optical design for the telecom range with the cutoff over longer wavelength which is a cause of the extrinsic dark count. The results presented in this paper show that this is a powerful technique for precisely tailoring the spectral dependence of SSPDs and will benefit a host of emerging multidisciplinary applications, including fluorescence spectroscopy in the life sciences, atmospheric remote sensing and secure communications.

## Additional Information

**How to cite this article**: Yamashita, T. *et al*. Superconducting nanowire single-photon detectors with non-periodic dielectric multilayers. *Sci. Rep.* **6**, 35240; doi: 10.1038/srep35240 (2016).
